# Peri-appendicular Abscess in a Spigelian Hernia

**DOI:** 10.1055/s-0042-1758044

**Published:** 2022-11-06

**Authors:** Rany Aoun, Rhea Akel, Roger Noun, Ghassan Chakhtoura

**Affiliations:** 1 Department of Digestive Surgery, Hotel Dieu de France Hospital, University Saint Joseph Medical School, Beirut, Lebanon; 2 Department of Radiology, Hotel Dieu de France Hospital, University Saint Joseph Medical School, Beirut, Lebanon

**Keywords:** acute appendicitis, Spigelian hernia

## Abstract

**Background** Spigelian hernias are a rare type of lateral ventral abdominal hernia and their content can include any of the intra-abdominal organs. Many cases have described the presence of a variety of abdominal organs in Spigelian hernias, but only few cases report the presence of an incarcerated appendicitis. Imaging is an important step in the diagnosis to avoid the lack of knowledge in such cases. Surgical treatment can be through open or laparoscopic approach, with or without using a mesh according to the size of the defect.

**Case Report** We report a case of an 82-year-old patient who presented with an acute appendicitis with peri-appendicular abscess strangulated in a right Spigelian hernia. The patient was successfully treated by a laparoscopic appendectomy, a surgical drainage of the abscess, and direct muscle approximation without using of mesh due to inflammation.

**Conclusion** Spigelian hernias with acute appendicitis in their content are a very rare condition. Clinical diagnosis is usually difficult and challenging and computed tomography scan is the imaging modality of choice. The treatment is surgical.

Abdominal wall hernias through Spigelian aponeurosis are a rare type of hernia. 1 Usually, it is a small defect in the fascia, and the clinical diagnosis is difficult unless there is incarceration or strangulation of their content. In this case, awareness of the content of the hernia is required to guide the management. Although many cases were described before, but the lack of knowledge is still present and can delay medical care. We present a case of a patient who presented with an acute appendicitis strangulated in a right Spigelian hernia.

## Case Presentation

We describe the case of an 82-year-old patient, suffering from chronic kidney disease due to diabetes, who presented to his dialysis session complaining of exacerbated abdominal pain located at the right flank, associated with shivers. He mentioned an abdominal discomfort, which started 5 days before with increasing intensity until it became unbearable. He denied experiencing nausea, vomiting, constipation, or diarrhea.

The patient, known to have chronic kidney disease, is on hemodialysis since 2019 through an arteriovenous fistula, three sessions per week. His past medical history is relevant for type 2 diabetes mellitus, long-standing hypertension, hyperlipidemia, hyperuricemia, heart failure (ejection fraction 35–40%), and atrial fibrillation. He has a 200 pack/year smoking history, which stopped in 2001. His medical history is also significant for recurrent hypoxic pneumonias, chronic obstructive pulmonary disease exacerbations, and recurrent pyelonephritis.

His surgical history includes left nephrectomy, exploratory laparotomy for penetrating gunshot wound, bilateral cataracts, and corneal transplants.

On physical examination, the patient is obese, conscious, and alert. Vital signs were within normal limits, with no fever. Body mass index is 31 kg/m ^2^ .

Bowel signs were present. Palpation of the abdomen revealed an indurated painful mass at the right flank. No redness or signs of cutaneous inflammation were seen on the abdomen. Otherwise, the abdomen was soft, with no rebound tenderness or guarding.

**Fig. 1:**
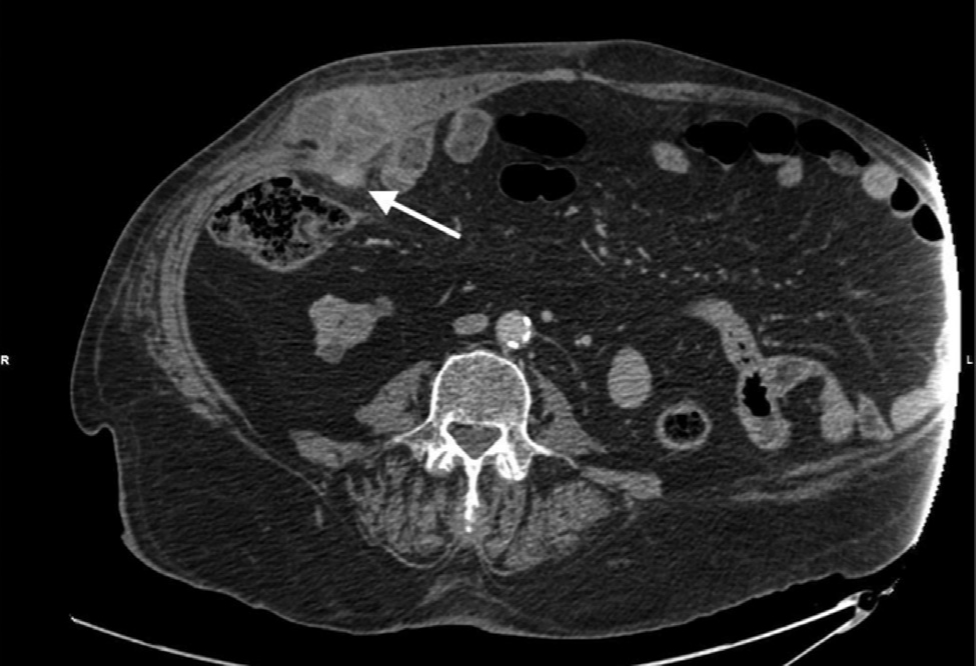
Computed tomography scan showing the tip of the appendix (arrow) protruding through the Spigelian hernia.

**Fig. 2:**
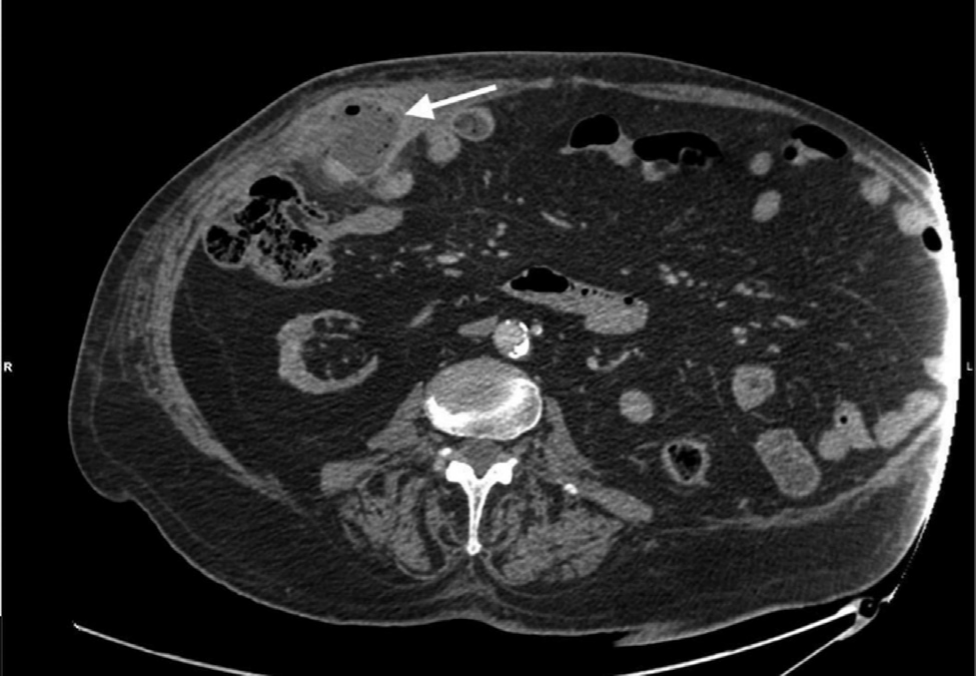
Computed tomography scan showing the peri-appendicular abscess (arrow) of 60 × 40 mm in the Spigelian hernia.

**Fig. 3:**
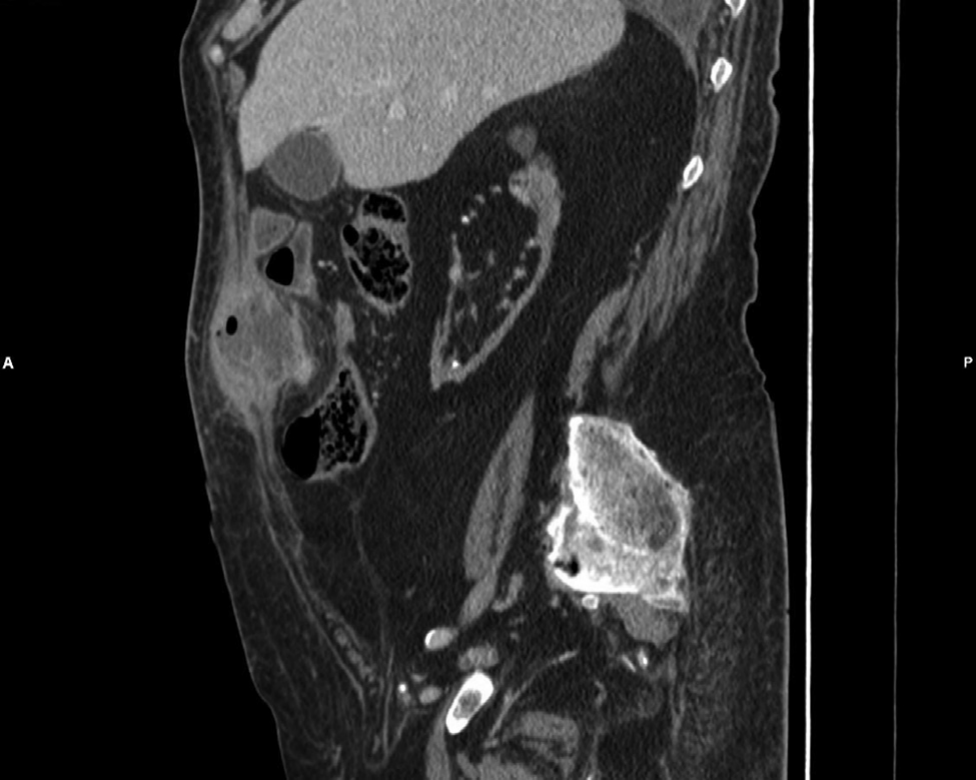
Sagittal view of the abscess.

A computed tomography (CT) scan of the abdomen with intravenous contrast showed an inflamed and thickened appendix, surrounded by fat stranding, with the formation of a loculated abscess of 60 × 40 mm at its tip, protruding through the muscle wall and strangulated in a right Spigelian hernia ( Figs. 1 - 3 ). Laboratory test results showed 12.6 × 10 ^9^ /L of white blood count and a protein C reactive level at 202 mg/L.

**Fig. 4:**
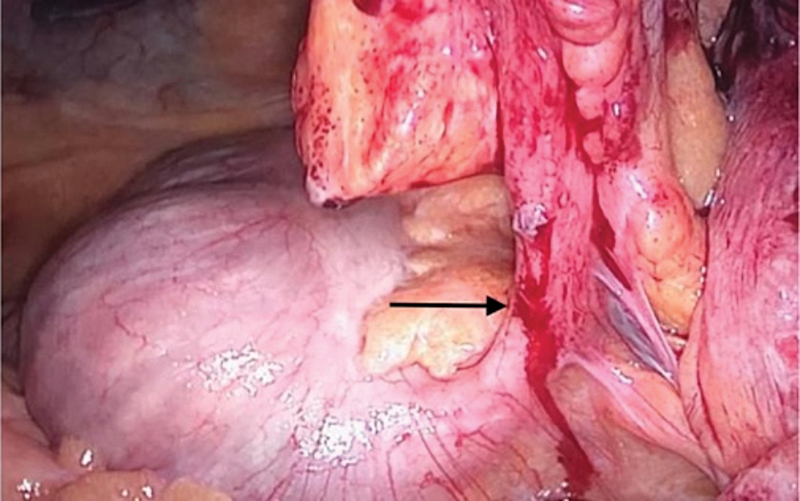
Perioperative view of the base of the appendix (arrow).

**Fig. 5:**
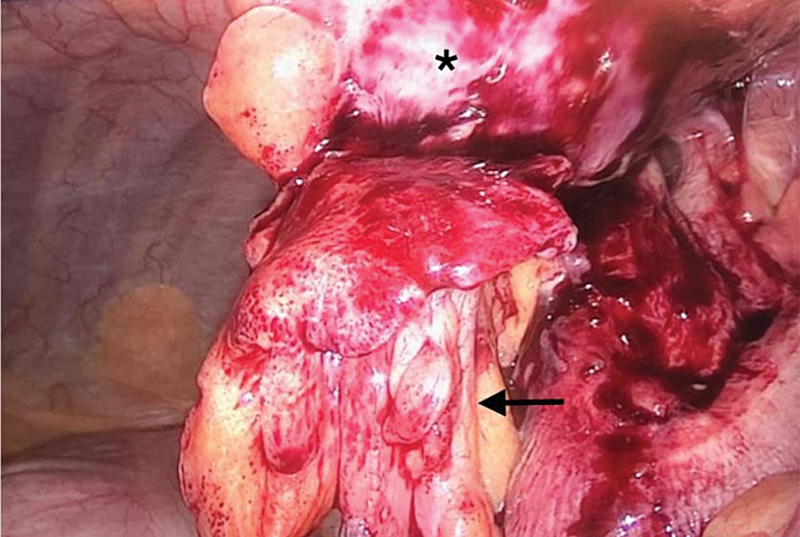
Perioperative view showing the appendix and its meso (arrow) protruding through the Spigelian hernia and the abscess bulging (asterisk).

An empiric large-spectrum antibiotic was initiated (Piperacillin-Tazobactam) since the patient has a long history of hospitalization and recurrent infections (urinary and pulmonary). He was then transferred to the operating room. The operation was a laparoscopic appendectomy (ligation of the base and controlling the mesoappendix) and a surgical drainage of the peri-appendicular abscess located on the right flank ( Figs. 4 and 5 ). Then, a direct repair of the external oblique muscle and its aponeurosis was performed by approximation with Vicryl 0 suture. The defect was smaller than 15 mm and we decided not to put a mesh. Although there was no redness or signs of inflammation, the subcutaneous cavity was left open and an impregnated gauze was left there for the drainage of pus and the contamination occurring during the operation. The patient was stable during the operation and the operative time was 1 hour.

Culture of the abscess showed the growth of *Escherichia coli* sensitive to the previously used antibiotic. There was no chance for downgrading the antibiotic. The patient had an uneventful hospital course and he was discharged on day 7. During this week, the patient received the same antibiotic for 48 hours and the gauze was changed daily. The pathology report showed an acute appendicitis with peri-appendicular abscess integrated within the mesoappendix and no signs of malignancy. Postoperative follow-up at 1 month revealed a recovered patient from this episode without any complaints.

## Discussion

Spigelian hernias are a rare type of abdominal wall hernia, with an incidence of only 1 to 2% of all hernias. They are more common in women, can be congenital or acquired, and predisposing factors, such as obesity and pregnancy, can play a role. In definition, a Spigelian hernia is a weakness in the aponeurosis located between the rectus muscle and the linea semilunaris. 1 All intra-abdominal organs can protrude through this defect; most common are the omentum and the small bowel. Reviewing the literature, only 10 patients experienced the same condition of an acute appendicitis strangulated in a Spigelian hernia. 2 3 4 5 6 7 8 9 10 11

It is always challenging to diagnose a Spigelian hernia without complications. The hernia is difficult to assess on clinical examination, especially in obese patients. In the absence of incarceration or strangulation, the patient describes a chronic diffuse abdominal pain, increased by contraction of the abdominal wall musculature.

When a high index of suspicion is found, sonography appears to be effective and accurate in the diagnosis. 12 In an emergency case, CT scan is more reliable to rule out any other intra-abdominal pathology, as in our case.

Chronic herniation caused adhesions and exteriorized the perforation from the abdominal cavity, preventing our patient from developing more severe medical conditions, such as peritonitis. Cox et al 6 also noticed this fact.

Surgical correction is the treatment for Spigelian hernias. A review of different surgical approaches in the cases discussed above appears in Table 1 . Different techniques were described: open and laparoscopic. The use of synthetic mesh is also discussed for patients with large defects. 13 Immunosuppressive drugs, urgent repair, and postoperative surgical site infection are predictive to mesh infection. 14 In our patient, we decided not to use a mesh for repairing the defect for the following reasons: the urgent character of the operation, the contamination of the surgical site by drainage of the abscess, the history of the patient who is suffering from chronic kidney disease and under hemodialysis, and finally the small defect.

**Table 1:** Surgical approach in the previous studies

Study	Surgical technique	Using of mesh
Thomas et al (2013) 2 ; Thomasset et al (2010) 3 ; Cox et al (2017) 6 ; Lin et al (2010) 7 ; Ramírez-Ramírez and Villanueva-Sáenz (2017) 10 ; Demetriou et al (2012) 11	Open	No
Deshmukh et al (2010) 4	Laparoscopic	Not mentioned
Onal et al (2003) 5	Open	Yes, polypropylene
Reinke and Resnick (2010) 8	Laparoscopic	No
Peeters et al (2017) 9	Combined laparoscopic and open	Yes, 2 months later
Our case	Combined laparoscopic and open	No

## Conclusion

Acute appendicitis strangulated in a Spigelian hernia is a very rare condition that presents with a painful indurate flank mass. CT scan is essential for diagnosis, surgical treatment is required, and laparoscopic approach is safe. More studies are a must to approve the use of mesh in this condition of hernia repair.
